# Bench Research Informed by GWAS Results

**DOI:** 10.3390/cells10113184

**Published:** 2021-11-15

**Authors:** Nikolay V. Kondratyev, Margarita V. Alfimova, Arkadiy K. Golov, Vera E. Golimbet

**Affiliations:** 1 Mental Health Research Center, 115522 Moscow, Russia; m.alfimova@gmail.com (M.V.A.); golovstein@gmail.com (A.K.G.); golimbet@mail.ru (V.E.G.); 2 Institute of Gene Biology, Russian Academy of Sciences, 119334 Moscow, Russia

**Keywords:** complex traits, GWAS, polygenic scores

## Abstract

Scientifically interesting as well as practically important phenotypes often belong to the realm of complex traits. To the extent that these traits are hereditary, they are usually ‘highly polygenic’. The study of such traits presents a challenge for researchers, as the complex genetic architecture of such traits makes it nearly impossible to utilise many of the usual methods of reverse genetics, which often focus on specific genes. In recent years, thousands of genome-wide association studies (GWAS) were undertaken to explore the relationships between complex traits and a large number of genetic factors, most of which are characterised by tiny effects. In this review, we aim to familiarise ‘wet biologists’ with approaches for the interpretation of GWAS results, to clarify some issues that may seem counterintuitive and to assess the possibility of using GWAS results in experiments on various complex traits.

## 1. Introduction

Complex traits, by definition, depend on a large number of genetic and environmental factors and the interaction between these factors. Complex traits are both the most common and the most interesting phenotypes to study. The key problem in the study of complex traits is the difficulty of researching them using reverse genetics methods, the purpose of which is to search for unknown functions of a molecular sequence by introducing mutations and tracking emerging phenotypes. Naturally, when the expected effect of each of the numerous genes on the phenotype is very weak, such studies are extremely difficult to conduct, as they involve multiple directional changes in the genetic apparatus of the cell that are not possible with current directed genome editing technologies. In addition, for complex traits, it is often unclear what specific changes should be made in cells to obtain the desired phenotypes.

This question can be answered by classical forward genetics, which aims to establish the genetic variability associated with the variability in the studied trait by, for example, searching for mutations responsible for a specific phenotype in screens conducted in model organisms using artificial mutagenesis. The field of forward genetics has been enriched with new tools in the last ten years owing to the development of new technologies: genome sequencing and hybridisation arrays. Single-nucleotide polymorphism (SNP) hybridisation arrays make it possible to perform experiments such as genome-wide association studies (GWAS) with simultaneous interrogation of the allelic status of hundreds and later hundreds of thousands of polymorphisms. Recently, thousands of GWAS have been carried out, the results of which can serve as a foundation for subsequent biological experiments. However, it is difficult not to notice the weak interest of experimental biologists in the results of GWAS. We believe that this is largely due to a cultural barrier between geneticists and experimental biologists. From the point of view of devoted experimentalists, GWAS are often perceived as overpriced experiments aimed at exploring the genetics of overly specific, reductionist or bizarre traits such as exhaustion in shift workers [1], restless leg syndrome [2], household income [3], colour of meat of Atlantic salmon [4], self-reported habitual walking pace [5], only to find a bunch of tiny effects that individually have almost no influence on a trait. Why these studies are completely acceptable—and they are—demands an explanation. The purpose of this review is to acquaint ‘wet biologists’ with what is happening on the other side of the barrier, why the data obtained in GWAS can be taken seriously and, most importantly, how it could be used in their experimental work.

## 2. GWAS Is a Major Tool for the Genetics of Complex Traits

**Figure 1:**
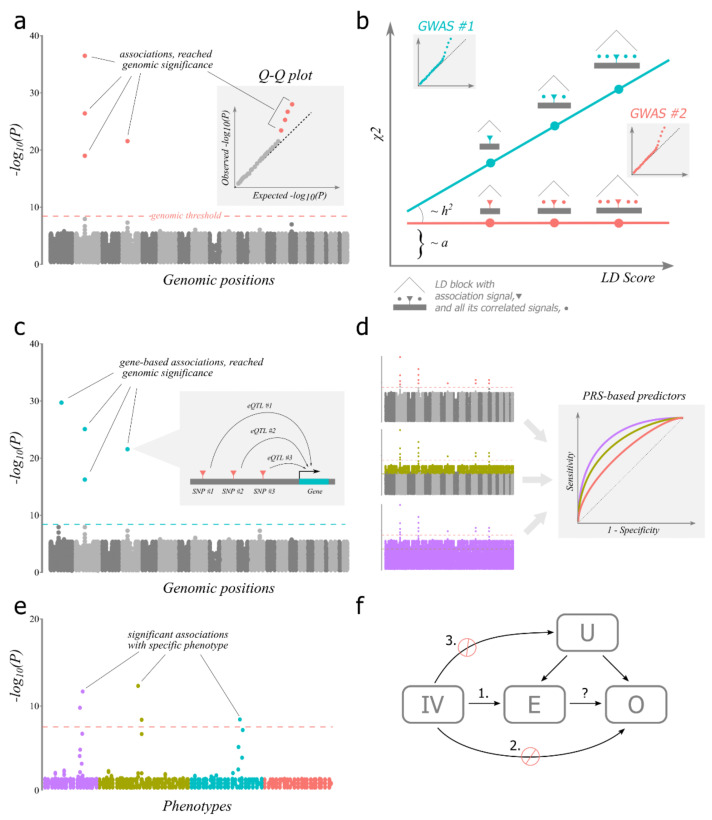
Main concepts discussed in the text. (**a**) *GWAS*. The scheme depicts the Manhattan plot, a main visualisation of GWAS results. Manhattan plot shows distribution of observed *p*-levels of individual association tests across genomic positions (represented as dots). Manhattan plot allows to quickly assess how many associations pass the genomic significance threshold (dashed line). Inset depicts the corresponding quantile-quantile (Q-Q) plot which shows the distribution of observed versus expected *p*-levels. (**b**) *LDSC*. The scheme shows differences in two GWAS experiments both with population bias and only one has true genetic effects (turquoise). The Q-Q plots for both experiments are depicted as insets to illustrate that they look the same. LD score is a sum of correlations between tested SNPs for a given SNP. Chi-squared is a measure of effect for a given SNP, modelled as a random variable. The slope of the regression is proportional to heritability (*h^2^*) and the intercept (a) is proportional to bias. (**c**) *TWAS*. The scheme depicts the Manhattan plot with gene-based associations. The inset shows how an individual association is produced with external eQTL data which are used to predict gene expression by genotypes in GWAS data. (**d**) *PRS*. The scheme demonstrates a typical situation in GWAS when polygenic scores calculated with relaxed genomic thresholds perform better than with strict threshold. Three ways of selecting associations from the same GWAS for PRS calculation are presented (left). ROC curves (right) represent predictive models based on PRS, calculated using significant (index) associations only (salmon), with relaxed threshold (olive) and all SNPs (‘omnigenic’, lilac). (**e**) *PheWAS*. Association tests of a broad range of phenotypes grouped by similarity for a specific genotype are depicted. The typical situation where similar phenotypes have similar degree of association is depicted. (**f**) *Mendelian randomisation*. The scheme shows the experiment of studying the possible causal relationship (an arrow, marked with the question mark) between exposure (E) and outcome (O) with instrumental variable (IV) and possible unknown confounders (U). Numbered are conditions for IV to be valid, stop signs symbolise forbidden relations.

The emergence of GWAS as a full-fledged experimental design has already been described in excellent reviews [6,7]. In short, at the end of the 20th century, the key method for identifying the genetic factors responsible for a studied trait was the analysis of the linkage of the regions of the genome co-segregated in families with this trait (‘linkage analysis’). This analysis was capable of identifying risk factors with strong effects, such as the association of the ε4 allele of the *APOE* gene with Alzheimer’s disease (AD) [8,9] and genetic risk factors for breast cancer in the *BRCA1* [10] and *BRCA2* genes [11]. However, linkage analysis was unsuccessful for most of the complex traits studied. A new approach that would allow the identification of genetic factors with small effects on polygenic complex traits was needed. Genome-wide testing of genotypes should rely on associations of genotypes with traits, and not on linkage analysis, since the former has significantly higher statistical power (Figure 1a) [12]. The ability to carry out genome-wide association analysis was one of the motivations for the Human Genome Project [13] and subsequent projects to study widespread inheritance in human populations, most notably the HapMap Consortium [14].

It is expected that variability that is causal for the complex trait under study will be detected experimentally based on the trait-dependent frequency of the marker alleles with which the causal genotype is linked. Since there are several hundred thousand independent linkage groups for polymorphisms in the human genome, in GWAS, genotypes for all of these polymorphisms must be obtained and tested for association with a trait. The direct result of GWAS is a list of statistically significant associations mapped to specific genome regions (‘GWAS linkage regions’ or ‘GWAS hits’). As we will see below, the complete GWAS results (i.e., what effects [their size, direction and statistical significance] were obtained for each of the studied polymorphisms) are equal if not more important, regardless of whether they reach the genome-wide significance level.

Performing GWAS implies that common genetic variation for an organism of interest is known beforehand. The typical linkage disequilibrium (LD) structure should also provide a sufficient (yet not extensive) number of tagging polymorphisms to capture genetic variability. In most cases, this requires efficient and cheap technology for mass genotyping. Though it was possible to perform large-scale genotyping with conventional methods [15], GWAS were kick-started in the late 2000s, when mass genotyping with hybridisation arrays became available [16,17]. Custom arrays were created for genotyping of many organisms, such as *Arabidopsis thaliana* [18], mouse [19] and Atlantic salmon [20]. Currently, Illumina provides specialised genotyping arrays for human, porcine, bovine, equine, maize, mouse, potato, ovine and Pacific white shrimp genetic research. The genetic information required for GWAS can also be obtained directly from DNA sequencing, which is a convenient method for organisms with smaller genomes, such as yeast [21] or *Caenorhabditis elegans* [22]. As a rule, the use of the conventional whole-genome sequencing for GWAS is overkill for organisms with large genomes, but it could be justified in some cases. First, it is useful when a researcher’s primary goal is to collect data on rare mutations, as was done in massive studies on the genetics of blood metabolite levels [23], early onset atrial fibrillation [24] and Lewy body dementia [25]. Second, sequencing is required if GWAS is coupled with research on an unknown genetic variation, as in the case of the genetics of sand pear fruit quality [26]. Finally, for organisms with extensively researched haplotype population structure, such as humans, it is possible to perform genotyping with low-pass full-genome sequencing with coverage <1 followed by imputation with reference haplotypes. The cost of genotyping with this method could be substantially less than with even the cheapest available genotyping arrays and will probably replace them as the principal genotyping method in the future [27,28].

**Figure 2:**
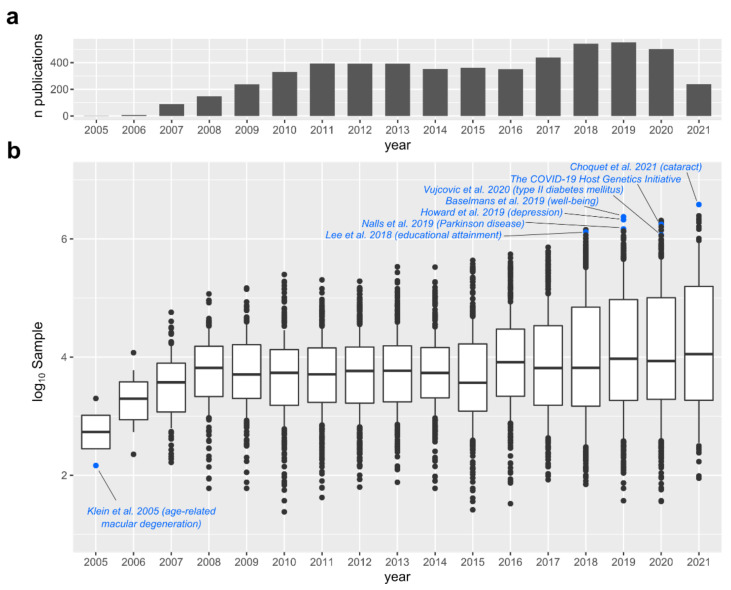
Published studies with available summary statistics that are included in the GWAS Catalog. (**a**) Number of studies added to the GWAS Catalog by year. (**b**) Sample sizes in the GWAS Catalog over time. Some studies mentioned in the text are highlighted. The data were accessed through the GWAS Catalog site on 21 September 2021.

The first conventional human GWAS appeared in 2005, when the genetics of age-related macular degeneration was investigated in a modest sample of 50 patients and 96 controls [29]. Thousands of GWAS have been performed since then, with the sample size of the typical GWAS growing over time to provide more statistical power for detection of smaller effects (Figure 2). The need for sufficient samples for GWAS led to an unprecedented degree of collaboration in complex traits genetics. Many consortia of researchers were created to achieve the goal, including the Genetic Investigation of ANthropometric Traits (GIANT) Consortium [30], the Psychiatric Genomics Consortium (PGC) [31], the DIAbetes Genetics Replication And Meta-analysis (DIAGRAM) [32], the International Parkinson’s Disease Genomics Consortium (IPDGC) [33] and many others. The efforts of hundreds of laboratories working together in these consortia allowed them to carry out research on an enormous number of samples. At the time of this writing, 31 of the studies in the GWAS Catalog had a sample size exceeding one million participants, though most of the large studies are meta-analyses. Some examples of the largest studies to demonstrate the diversity of human phenotypes studied with GWAS include those focusing on: (1) Physiological traits such as blood pressure [34], cholesterol level [35] and concentration of liver enzymes in blood serum [36]; (2) medical conditions such as breast cancer [37], chronic renal failure [38], osteoporosis [39], Parkinson’s disease [40], diabetes [41], cataract [42] and dental caries [43]; (3) anthropometric traits such as height [44], longevity [45], handedness [46], body fat distribution [47]; (4) lifestyle traits such as alcohol consumption [48], smoking [49] and chronotype [50]; (5) psychological traits such as self-reported depression [51], risk tolerance [52], intelligence [53], well-being [54], ‘Big Five’ personality traits [55] and even (6) socioeconomic traits such as educational attainment [56], family income [3] and being fired from work [57]. It is difficult to find a common human phenotype that has not yet been studied with GWAS.

It is a rare situation when an analytical framework is developing primarily for a human model, especially for anthropometric traits and psychiatric and common autoimmune diseases. However, non-human GWAS are also developing rapidly. Non-human models could provide unique advantages for complex traits genetics research. For organisms that can be maintained as isogenic lines (for example, those capable of self-fertilisation), it is especially convenient to conduct GWAS for new traits with preliminarily genotyped collections of specimens [58,59]. For example, such collections have been generated for *A. thaliana* [18], sorghum [60], *Drosophila melanogaster* [61], rice [62] and many others. When extreme genetic transgression can be achieved, the collections of natural variation are not even required, as it is possible to easily create artificial genetic and phenotypic variation using genetically diverse strains, as can be done with yeasts [63], *C. elegans* [22] and even mice [64]. For some species, it could be beneficial to create a synthetic population with interspecies hybrid crossing, as was done in a study of hypoxia in catfish [65].

In human genetics, raw genotyping data for specific individuals are almost never available to unauthorised researchers. However, to reuse GWAS results, it is often enough to have access to published *GWAS summary statistics* (i.e., information about allelic effects and statistical significance over all tested polymorphisms relative to a studied trait). However, many studies do not report summary statistics, although the situation is improving [66]. The severity of the problem depends on the field; for example, the share of public GWAS summary statistics in oncology is currently the lowest among all of human biomedical research [67]. Currently, the UK Biobank stands out from the large individual research centres, as it provides access to GWAS results for 4541 (at the time of the writing) different biomedical, psychological and socioeconomic phenotypes with freely available and harmonised summary statistics files. In general, the database of large-scale genetic research (NHGRI-EBI GWAS Catalog) [68] has, at the time of writing, 5329 records of published studies for over ten thousand traits. For other organisms, the universal resources of GWAS data are not yet available, with the noticeable exception of *A. thaliana* genetics: the AraGWAS Catalog currently contains data for 462 phenotypes from approximately 20 individual studies [69].

## 3. Assumptions of GWAS

### 3.1. Heritability of Complex Traits

GWA studies are often referred to as ‘hypothesis-free’ research, as opposed to the classic ‘candidate’ association analysis, in which the researcher initially assumes a link between a specific locus and a disease. The hypothesis-free approach to genetic research has a number of advantages; in particular, it allows the discovery of new unexpected genes associated with a trait, thereby acting as ‘hypothesis-generating machine’, and is not subject to the effect of overrepresentation of positive results in the literature (publication bias) characteristic of candidate studies [70]. Nevertheless, there is a hypothesis at the heart of GWAS: that a complex trait has a specific hereditary nature. A typical genetic experiment is devoted to the study of diversity associated with heredity. It is often possible to know in advance what proportion of the variability of a trait is determined by genetic factors. This number, the *heritability* of the trait, can be determined from, for example, twin or adoption studies. Estimates of the heritability of a trait are useful for planning a GWA study—the lower the heritability, the more samples needed for a successful experiment [71]. Note that very often our intuition about the role of genetics in a trait is wrong, especially when it comes to behavioural and socioeconomic traits [72].

It is important to note some properties of heritability. First, heritability is not a measure of how much a trait is determined by genes; rather, it indicates how much the variability of a trait is explained by genetic variation in the studied population. Variability may be low and heritability high, as in the case of schizophrenia, which, despite being a ‘common disease’, still affects only a small fraction (around 1%) of the general population. Conversely, for traits correlated with fitness, for example, such as fecundity and longevity, variation is big, but heritability is low [73]. Second, heritability is not constant, and it depends on the specific population in which it is measured. For example, a study of the heritability of tobacco dependence in the Dutch population at the beginning of the 21st century showed that it is as high as 0.75 [74]. However, such a study would have made no sense until the Dutch became acquainted with tobacco in the 17th century. On the other hand, it can be assumed that estimates of the heritability of adult literacy in the late 19th century were higher than they are today, since the variability in adult literacy in most countries has been reduced to zero by modern secondary education systems. This does not apply to literacy in preschool children, among whom there is indeed such variability and measuring the heritability of this trait makes sense. In a joint twin study on populations in Australia, Norway, Sweden and the United States, the heritability estimate for literacy in preschoolers was approximately 0.7 [75].

Modern GWAS with negative results in large samples are relatively rare. This led to the practice of performing GWAS without having preliminary data on the heritability of a trait. For example, genetic susceptibility to COVID-19 was quickly explored as early as in 2020 without prior understanding of whether heredity plays a role in the spread of the new virus causing this disease [76,77,78]. While this practice seems questionable, it should be noted that the GWAS results themselves could be employed to estimate the heritability of the trait (*h^2^*(SNP), heritability in the narrow sense or the theoretical limit of heritability, which could be explained by additive common variation; see the Section 3.3 below).

### 3.2. Population Structure

In a genetic experiment, the difference in the distribution of allele frequencies depending on the studied phenotype may be associated not with the phenotype, but with the unequal population structure relative to a trait, leading to false positive associations. The problem is more serious in small-scale genetic experiments, however. Massive genetic data often already contain controls for population structure, which can be obtained using principal component analysis or similar methods. A widely used practice in population genetics is to use genetic markers to map DNA samples from known genetic information. The accuracy of this method is even sufficient for use in forensic medicine. For example, the data on genetic markers of African elephants were enough to establish the specific site of poaching activity using DNA isolated from confiscated ivory [79]. The accuracy of genome-wide genetic data is even higher. Principal component analysis of genotypes even allows, for example, the reproduction of the main outlines of the geographical map of Europe from the genotypes of European ethnic groups [80]. When studying human populations, owing to the available reference panels of genotypes, such as International HapMap Consortium [14], 1000 Genomes Consortium [81] or The Haplotype Reference Consortium [82], it is easy to determine which sample belongs to which ethnic group. Thus, in a GWAS experiment, the data itself contain a control for the population structure.

A rigorous method for controlling population structure was developed based on the difference between how linkage disequilibrium relates to the true effects and to the effects caused by uneven population structure. The effects associated with uneven distribution of alleles due to differences in population structure should not, in contrast to true associations, depend on the length of the region with SNPs in high linkage disequilibrium (‘LD blocks’). It can be assumed, at least in the first approximation, that the position of causal polymorphisms for a trait under study is not related to how genotypes were recombined in the past; that is, the position of causal polymorphisms is random relative to the genetic recombination map. This means that the larger the LD block, the greater the chance of finding a higher level of significance for that region. If the effects identified are associated with segregation of haplotypes due to differences in population structure, the association signal should not depend on the length of the linkage region in which it is located (Figure 1b). The method based on this idea is called *LD score regression*, or LDSC [83].

LD score regression turned out to be one of the most influential ideas in the analysis of GWAS results. The use of methods based on LD score regression, as we will see below, allows researchers not only to control the population structure, but also to determine heritability according to GWAS data, the contribution to the heritability of a trait by functional classes of polymorphisms and the relationship of different traits to each other, as well as meet other objectives.

### 3.3. Common Additive Variation

The important assumption of GWAS is that the genetics of the trait under study is largely associated with common polymorphisms. Conceptually, when the fitness of an organism is associated with a complex trait, genetic factors with small effects have a great chance of avoiding the effect of negative selection, thus shifting the genetic architecture of the trait towards greater polygenicity with more common genotypes [84,85]. This was tested experimentally in the yeast model, and indeed, rare mutations with large effects were found to be more likely to be recent variants [86].

This does not mean that rare mutations with large effects do not affect complex traits; on the contrary, for many complex traits, such rare mutations with large effects have been found. The distinction between rare and common mutations is arbitrary and motivated largely by the methods used to explore these two types of variation. For example, it was found that common and rare mutations contribute to the variability of a trait in an additive manner for autism spectrum disorder [87] and obesity [88]. However, for a number of such traits (for example, coronary artery disease, type II diabetes and breast cancer), using GWAS has led to a situation in which comparable risk groups can be identified in a much larger number of people than can be identified using well-known mutations with large effects [89]. Moreover, GWAS for many traits with ‘simple’ genetics such as rare monogenic diseases were able to reveal additional genetic factors that can influence the severity of the disease. Such factors were identified in, for example, sickle cell anaemia [90], cystic fibrosis [91], acne [92] and Huntington’s chorea [93]. In fact, it is likely that all genetic traits are complex to some extent and can be studied with GWAS.

GWAS suggests that the underlying inheritance of the trait studied by GWAS should be mainly additive. This often seems counterintuitive to an experimental biologist, as it is natural to expect that the multilevel regulatory pathways characteristic of biological systems should be reflected in the genetics of a trait as epistatic interactions. Thus, it seems strange that it is difficult to find an example of clear epistasis in GWAS results. This could be explained by the fact that, in GWAS data, a weak statistical signal from non-additive interactions will be difficult to detect with a simple search due to the curse of dimensionality. Even for simple two-level interactions for N genotypes, there are (N^2^/2 − N) combinations times four types of epistatic interactions. The search for an optimal methodological approach aside from simple enumeration, including using machine learning and/or external functional data, has been the subject of a large number of studies (see reviews [94,95]).

However, finding multilevel epistasis in GWAS data can be challenging for reasons beyond statistical limitations. Even all of the people who have ever lived on earth are hardly enough to label all of the combinations of even a two-level epistasis for all LD-independent human polymorphisms. Intuitively, when a complex trait affects fitness, the evolutionary process will also be limited in the selection of non-additive effects. The defining importance of additive variability for evolution is emphasised in classical works on evolutionary biology: Fisher’s fundamental theorem postulates that the rate of evolutionary change is proportional to the additive genetic variability [96,97]. Conversely, epistatic effects are more likely to be found if variation of a complex trait violates assumptions of Fisher’s fundamental theorem. Indeed, in populations existing under artificial selection, like domesticated animals, epistatic effects can still be quite high [98,99]. The other case in which epistatic effects are observed is when a trait does not undergo selective pressure at all. It could be argued that human neurodegenerative diseases could be considered such traits, since they manifest well beyond reproductive age. Curiously, one of the best-known examples of epistasis in human GWAS data is the genetic interaction of the *KHDRBS2* and *CRYL1* genes in GWAS of AD [100]. In any case, it is unlikely that any complex trait will have genetics completely unrelated to fitness due to high polygenicity and pervasive pleiotropy of genetic factors of complex traits (see Section 4.4 below).

How functional interactions are reflected on a genetic level was tested directly in a 2021 study by Sinnott-Armstrong et al. In this study, GWAS was performed using data from the UK Biobank on metabolic traits (uric acid, IGF-1 and testosterone concentrations) that were deliberately selected to assess how consistent the GWAS data were with well-studied biological traits. As expected, most of the GWAS signals were found in the genes of the known biochemical pathways for the respective substances. However, epistatic interactions even between genetic factors associated with genes in the same biochemical pathway were either not detected or made a negligible contribution to the heritability [101]. It is possible to determine the importance of epistasis for the variability of complex traits by studying the genetics of organisms with small genomes. Yeasts are a convenient model for this type of experiment, since they allow the creation of a synthetic population for genetic testing with crossing of unrelated strains and phenotyping of easily measured traits like growth in the presence of various substances. This artificial system, simulating GWAS conducted under ideal conditions, revealed that even when the experiment has the necessary power to detect epistasis, additive genetic effects still make a decisive contribution to the heritability of a trait [63,86,102]. In this model experiment, the estimate of the contribution of non-additive heritability is probably even higher than expected in natural populations, since only the descendants from the first crosses were studied. Theoretical models predict that in natural populations, the contribution of epistasis to the variability of complex traits is even lower [103].

The underrepresentation of non-additive effects in GWAS data is an important feature of complex traits genetics. Without it, GWAS results would be substantially more difficult to interpret, compare and use in practice.

## 4. Arguments for GWAS

### 4.1. Reproducibility

GWA studies are characterised by high reproducibility in independent experiments. They tend to be carried out by large consortia that can bring together most of the scientists interested in studying a given trait. Therefore, the largest study in the field is often the only GWA study of comparable size, with most of the previously identified effects being reproduced at a new level [104]. An example of an impressive replication of the results of comparable GWAS can be found in recent genetic studies of vulnerability to COVID-19, as major independent studies found broadly the same loci associated with an increased risk of coronavirus infection [76,77,78]. The UK Biobank provides alternative GWAS results for a wide range of human traits, which allows the determination of how well the GWAS data from the UK Biobank reproduces previously conducted independent GWAS. Such an analysis was conducted on pairs of independent studies for nine traits and, in general, for polymorphisms that reached the genomic threshold in GWAS, the reproducibility was 85%. For stricter thresholds limiting the selected polymorphisms, reproducibility was even higher, with increases in both statistical significance and effect size [105].

It is normal for the same results to be obtained in studies on different human superpopulations, as evidenced by, for example, comparing GWAS on samples of people of European descent with GWAS on samples of people of East Asian or sub-Saharan African descent. The effects found for the same polymorphisms in such experiments are usually co-directional [106]. Moreover, associations with phenotypes are often located at the same loci for populations in which there are no same polymorphisms. Examples include height [107], blood lipid levels [108], type II diabetes [32,109,110], myopia [111] and schizophrenia [112]. In addition, in a new population, local genetic variability may be more advantageous than in an already tested one, as the new population may offer more common polymorphisms and a different linkage region. For example, the polymorphisms responsible for the association of the *NOD2* gene with Crohn’s disease in Europeans are absent in East Asians, and these important associations could not be found in GWAS in this population [113]. Such effects allowed the creation of a new experimental design of the GWAS, trans-ethnic GWAS, which has several advantages over the traditional approach: in particular, it allows more accurate mapping of causal variants [114,115].

For some traits, even interspecies similarity of the GWAS results can be observed. For example, there is strong overlap between the top GWAS results for human growth and the size of some mammalian domestic animals. Associations in the genes *LCORL* and *HMGA2* are in the list of GWAS signals for human height [116], as well as the size of dogs [99], cattle [117] and horses [118]. The use of GWAS to study the genetic causes of increased susceptibility to type II diabetes in Burmese cats has identified the same *ANK1* risk gene [119] as that identified in the corresponding study in humans [41]. GWAS for granulomatous colitis in a small sample of boxers and bulldogs identified a single signal in a region [99] for which a homologous region was identified in the human GWAS for inflammatory bowel disease [120].

It is also important to note the reproducibility of genetic effects at the level of the mutation spectrum. The genetic effects found in GWAS are typically small and in general manifest themselves on a regulatory level, while mutations with large effects act on the gene structure directly and are relatively rare. It seems natural to expect that both types of genetic factors are present at the same loci, and for some well-powered studies of both rare and common variations, this indeed turned out to be the case. Examples of traits for which this was observed include height [121], inflammatory bowel disease [122], type II diabetes [123] and schizophrenia [124]. In general, it was demonstrated that rare mutations with significant associations (calculated on a gene level) are enriched by GWAS signals in a large study of exome sequencing in UK Biobank samples [125].

Such effects explain the very well-known cases of comorbidity between monogenic and common diseases. GWAS signals tend to be enriched in the genomic loci linked with Mendelian diseases [126]. Genetic signals for phenotypically matched monogenic and complex traits (for example, growth defect syndromes and height, monogenic mood disorders and schizophrenia and Mendelian cardiovascular diseases and cholesterol level) were found to be enriched in the same loci at a significant rate [127]. It is sometimes possible to use this information to assist in the interpretation of GWAS results. For example, a genome-wide significant SNP associated with coronary artery disease resides in the promoter of the *LIPA* gene, mutations which are involved in the Mendelian diseases: Wolman disease and cholesteryl ester storage disease. A more complex example is the case of SNPs associated with body mass index (BMI), which through the use of Hi-C data (genome DNA interactions) could be linked to the relatively distant (>100 kbp) *CYP19A1* gene, which is phenotypically matched with BMI and involved in aromatase excess syndrome [127].

### 4.2. Interpretability

The strongest signals in GWAS often correspond to ‘natural’ biological interpretations. Examples include serum calcium concentration, corresponding to the calcium-sensitive receptor gene CASR [128]; dog size and the gene for insulin-like growth factor, IGF1 [99]; exhaustion in shift workers and the gene encoding melatonin receptor [1]; alcohol consumption and the gene for alcohol dehydrogenase, ADH1B [48]; number of cigarettes smoked per day and the gene encoding one of the nicotinic acetylcholine receptor subunits, CHRNA5 [49]; colour of meat of Atlantic salmon and the gene for beta-carotene oxygenase, bco1 [4]; and the anosmia symptom in COVID-19 and the genes encoding the odorant metabolising enzymes UGT2A1 and UGT2A2 [129]. However, most of the GWAS signals have unclear underlying biology, which motivates ‘post-GWAS’ experimental studies.

As with other ‘big’ biological data, there are formal ways to generate functional interpretations of GWAS results. It can be done simply by assigning SNPs to their functional categories individually, which could be enough to yield interesting results. For example, the analysis of early GWAS revealed that GWAS SNP hits are enriched in SNPs that are linked to regulation of gene expression—*expression quantitative trait loci* (eQTL) [130,131]. The enormous work of fitting cell-specific chromatin markers of ENCODE data to almost 1000 of the biggest GWA studies from the GWAS Catalog provided an expected picture of the distribution of traits to their corresponding affected tissues [132]. A more stringent approach is to consider the LD structure of GWAS data. There are a plethora of such methods that can be applied to GWAS summary statistics. Among the most popular are INRICH [133], DEPICT [134] and MAGMA [135]. The alternative method is to investigate the variability of a trait that could be explained by the variability of SNPs, or the portion of narrow-sense heritability, *h^2^*, that was captured in GWAS, *h^2^*(GWAS). It is possible to compute the contributions to *h^2^*(GWAS) of different groups of SNPs that are linked to a specific functional category. This idea was used to show a remarkable enrichment of genetic effects with DNase I hypersensitive sites, markers of genomic regions involved in transcription regulation [136]. Modern methods for partitioned heritability analysis are based on LD score regression (stratified LDSC, S-LDSC). Since the slope of LDSC regression is proportional to *h^2^*(GWAS), LDSC is able to correct for relatedness and population structure; in addition, the use of this method does not require access to raw genotypes, as summary statistics are sufficient [137].

These methods provide an interface between genetic epidemiological data and molecular phenotypes, such as cell-specific gene expression, active chromatin markers, DNA methylation and chromosome interactions, and allow one to assess the enrichment of genetic associations with various functional ontologies. These methods can be used to demonstrate enrichment of specific gene ontologies, like neurogenesis and locomotor behaviour in restless legs syndrome [2], cytokine signalling pathways for COVID-19 [138], cell growth and synapse organisation for volume of lateral nuclei [139] and cell adhesion and transsynaptic signalling for Tourette syndrome [140]. Hormozdiari et al., applied S-LDSC on sets of functional SNPs (eQTLs, etc.,) and found that they are indeed enriched for heritability in an expected, cell type-specific manner [141]. Surprisingly, similar results could easily be obtained with just conventional expression data and other functional data [142]. S-LDSC methods were used to demonstrate enrichment of genetic associations in brain cells, primarily pyramidal and medium spiny neurons of the cortex, in schizophrenia [143,144]; in several brain regions in GWAS of education attainment [56], risk tolerance [52] and household income [3]; in skin cells, particularly melanocytes, for melanoma [145]; in kidneys for serum urea concentration [101]; and in lungs for COVID-19 [78]. More elaborate methods have been used to obtain unexpected results. For example, the H-MAGMA method, a modification of MAGMA for working with chromatin interactions (Hi-C) data [146], was used on autism spectrum disorder GWAS data to identify a number of genes expressed in prenatal human brain [147].

Reference eQTL information allows the integration of GWAS data with specific genes to directly test for gene-based associations. Such an experiment is called a *transcriptome-wide association study*, or TWAS (Figure 1c) [148,149]. The same approach could be realised with information about the link between genetics and any other functional data (metabolite and protein levels, CpG methylation, splicing, etc.,), but expression reference data are usually much more available. TWA studies could provide insight into which specific genes in a specific cell context are involved in the biology of a trait, provide knowledge of directionality of an effect of gene expression on a trait and, as in typical differential expression studies, be analysed at the gene-set level to reveal underlying biological processes. TWAS could be performed using GWAS summary statistics with tools such as Fusion [149] and S-PrediXcan [150]. However, TWAS have several flaws compared to GWAS, related mostly to imperfections in eQTL reference panels (reviewed in [151]) and the inability to adequately capture eQTL effects *in trans* (see Section 5.2 below). With eQTL data available for an increasing variety of cell types and tissues via, for example, the GTEx Consortium [152], many traits have already been studied with TWAS, yielding many candidate genes for follow-up studies [153].

### 4.3. Utility

In principle, GWAS results could provide direct utility via identification of a successful candidate target for a manipulation of a trait. For example, GWAS of drought tolerance in maize yielded a signal in the ZmVPP1 gene, overexpression of which grants the plant drought tolerance [154]. In human genetics, GWAS results could be used to identify promising biomedical targets for common diseases, as many existing drug targets were retrospectively identified as association signals in GWAS (reviewed in [6,155]). Probably the cleanest example of new therapy emerging primarily from GWAS results is the prospective use of deucravacitinib for auto-immune diseases [156,157]. This substance acts on TYK2, the gene for which harboured the association signal in the early GWA studies for lupus [158], type I diabetes [159], psoriasis [160] and Crohn’s disease [161].

Such examples of direct GWAS utility are still relatively rare, but GWAS results are becoming more useful in predicting complex traits by means of genetic data via *polygenic risk scores* (PRS). The story of PRS is linked to the notorious ‘missing heritability’ problem; taken together, significant signals from early GWAS explained a negligible portion of heritability of a trait (*h^2^*(GWAS) << *h^2^*) [162]. Later it became clear that heritability, explained by common variation, was not ‘missing’, but ‘hidden’ due to the fact that the polygenicity of the studied traits turned out to be much higher than previously expected [163]. This was first discovered in one of the first GWAS for schizophrenia. Schizophrenia is a common disease and the heritability of schizophrenia, as determined in twin studies, is quite high, so it is reasonable to expect that GWAS on schizophrenia would explain much of this heritability. One can imagine how disappointing the results of the first GWAS for schizophrenia were [164,165,166], as these enormous efforts led to the discovery of only a few significant linkage regions that in total explain a minuscule part of the heritability of the disease.

In one of these GWAS, a previously proposed methodology [167] was used to calculate a generalised number that characterises the genetic risk of a disease as the sum of allelic effects found in GWAS. The calculation included all LD-independent polymorphisms for which a certain threshold level of significance was reached, including thresholds that were less stringent than the genomic threshold of significance. The number calculated using this method was called the polygenic risk score (sometimes called the genome-wide polygenic score and abbreviated as GPS). This PRS turned out to have a peculiar feature: it approximates the risk of the disease much better if a relaxed threshold level of significance is used to calculate it. That is, most of the polymorphisms that determine the risk of disease do not cross the genomic threshold for GWAS (Figure 1d). It is worth noting that all of these conclusions were made on the basis of the PRS, which then explained only about 3% of the variability. Nevertheless, this was still an order of magnitude more than the share of heritability explained by only the significant GWAS hits [164]. Notably, modern techniques for PRS calculation like LDpred2 [168] use advanced procedures for defining independent SNPs and for association threshold optimisation, but the performance is not drastically better than the original approach.

This can be interpreted as follows: (1) Most genetic mutations are linked in some way to functional mutations that affect the development of the disease; (2) for most of these mutations, the effects are too small to be reliably established in a GWAS performed on a sample of a realistic size; and (3) the contribution of the totality of such small genetic effects to *h^2^* outweighs the contribution of mutations with strong effects. This is likely due to the fact that biological systems are able to respond even to mutations that are weakly associated with causal biological mechanisms. This view has been articulated in the ‘omnigenic hypothesis’ of common diseases [169,170]. In the case of schizophrenia, a disease of the brain, this assumption seems realistic, since approximately a third of human genes are associated with the functioning or development of the nervous system (for example, the CL:0002319 ‘neural cell’ ontology group now includes more than 5000 human genes, and GO:0007399 ‘nervous system development’ includes more than 6000 genes), and in each typical linkage group, there is either such a gene or a genomic region (e.g., enhancer) that controls the expression of the gene.

Although polygenic scores were originally used in psychiatric genetics, this methodology has become the standard for interpreting the results of any GWAS. The fact that polygenic scores explain significantly more variability in the trait than the sum of all statistically significant GWAS results has been repeatedly confirmed for other types of complex traits. This holds true even for the most powerful GWAS to date. For example, 3290 significant GWAS hits for human height with a sample size over 700,000 people together explained 24% of the variability in height, while PRS accounted for 34.7% [44]. A previous GWAS on height that included roughly 250,000 individuals estimated 10% of the variability in height, explained by 697 significant associations, while PRS accounted for 29% of the variation [116]. Curiously, such genetic complexity is not consistent across species. As an extreme example, 83% of the phenotypic variability in horse size is attributed to just four common genetic factors—no doubt a consequence of vigorous artificial selection [118].

While more powerful studies could resolve some of uncertainty about previous weak GWAS associations, it is doubtful that any substantial increase in sample size would yield a substantial increase in the variability explained by significant associations. Likewise, more sophisticated analysis might not help either. There have been multiple attempts to build more complex predictive models based on whole-genome genetic data, rather than relying on the sum of weighted effects with simple *p*-level thresholding as SNP (feature) selection. It is remarkable that such approaches appear not to have significantly improved PRS prediction to date [171,172,173,174]. As discussed above, this could be due to a lack of substantial non-additive (epistatic) effects for a typical complex trait.

However, in many cases, PRS are already the best-known predictors for a complex trait. An example is the use of PRS to identify individuals at increased risk for various common inherited diseases: type II diabetes, inflammatory bowel disease, breast cancer, atrial fibrillation and coronary artery disease [89]. For coronary artery disease, the results were particularly impressive, as the PRS predictor was found to be more accurate than the cumulative genetic markers already used in current clinical practice [89,175]. In a study conducted on a sample of 47,000 people, of which 11,000 were diagnosed with coronary artery disease, it was demonstrated that people who fall into the upper quintile for polygenic risk have twice the risk of coronary artery disease compared to the general population [176]. Examples of studies on the use of PRS in predictive models of disease risk include those evaluating the effectiveness of PRS in predicting breast cancer [177,178], prostate cancer [179], glaucoma [180] and longevity [181]. A study by Zhang et al., focused on what predictive performance could realistically be expected for PRS for 14 types of cancer. They estimated that the range of performance lies between AUCs of 0.63 and 0.89, with the lowest estimate for ovarian cancer and the highest for testicular cancer [182]. In a massive exome study of obesity, it was shown that the prevalence of obesity on a background of risk-increasing mutations in the *MC4R* gene and protective mutations in *GPR75* are greatly modified by PRS of BMI, with an absolute difference in prevalence of approximately 60% between individuals belonging to extreme PRS quintiles [88]. The inclusion of PRS in predictive models for the transition to AD in individuals with mild cognitive decline improves the model compared to models based on *APOE* alone, with the AUC increasing from 0.68 to 0.84 [183,184]. For Parkinson’s disease, the best models currently predict the disease at an AUC only up to 0.65, which indicates that this is a poor predictor [40,185]; however, stratification by PRS allows the identification of the time of disease manifestation [186].

An impressive result demonstrating the predictive power of PRS was obtained in a GWA study on education (‘educational attainment’ and ‘educational achievement’) [56]. It would seem that a complex social trait like education would be difficult to predict based on genetic data. Nonetheless, this study turned out to be one of the most successful GWAS, largely due to the massive sample of more than one million people. Later, it turned out that this PRS explained approximately 15% of the variability in the duration of education, correlated (*r* = 0.4) with students’ final grades [187] and predicted educational success almost as well as the best predictor, family’s socioeconomic status [188,189]. One study even showed that genetic differences are entirely responsible for the difference in learning outcomes in UK schools both with and without pre-selection of students [190]. We should note, however, that the utility of these results is mainly in informing effective education policies as opposed to using education PRS on an individual level (see [191]). Prediction of education PRS is partially related to heritable family environment, as evidenced by the drop in the predictive power of PRS for education in adopted individuals [192]. This illustrates that just as for heritability estimates, polygenic scores do depend on the original population in which they were calculated.

The use of PRS can significantly improve the prediction of the outcome of a pleiotropic rare mutation. For example, carriers of a 22q11.2 deletion (DiGeorge syndrome) are developmentally delayed, but in addition to this, approximately 20–25% are at risk of developing schizophrenia [193,194]. The PRS calculated for schizophrenia significantly modifies the risk for carriers of the mutation: if the polygenic risk for schizophrenia is taken into account, then the risk of developing schizophrenia for carriers of 22q11.2 deletions is 33% for the highest decile and only 9% for the lowest decile. Similarly, for lack of intellectual development (measured as IQ < 70 with an a priori risk of approximately 40%), if PRS for IQ is used, then the risk of decreased IQ is 63% for carriers from the lowest decile and 24% for carriers from the highest decile [195].

When using a PRS, one should consider differences between the target population and the population in which the GWAS summary statistics used to compute the PRS was determined. For example, the average prediction accuracy for 17 quantitative traits, calculated for the European GWAS, falls approximately four-fold for the sub-Saharan African population [196]. Note that while PRS have low generalisability in ‘non-native’ populations [197], the use of PRS derived from trans-ethnic GWAS seems to provide an advantage over single-origin GWAS, even in a ‘native’ context [198].

Unfortunately, for many complex traits with low heritability and/or low phenotypic variation, it is impractical to achieve a sample size necessary to reliably establish genomic loci responsible for the majority of the heritable phenotypic variation. It seems that for many of the important complex traits, PRS remains the best measure to approximate genetic predisposition. Fortunately, PRS, though a simplified model of genetic risk, still often proves to be a quite effective complex trait predictor, often better than those already existing or at least able to produce a substantial increase in performance in a joint application.

### 4.4. Interoperability

Important evidence supporting GWAS is that genetic factors of similar complex traits tend to be similar themselves. This would of course not be the case if GWAS effects were just random noise. We have already noted that genetic factors for some monogenic traits are often found in the same genomic regions identified in GWAS for similar complex traits. While more powerful methods for estimation of co-heritability use individual genotype information [199], it is possible to employ the summary statistics information from GWAS (reviewed in [200] with, for example, LDSC [201,202,203]).

With more data available for different traits, the study of pleiotropy became more common and can be exploited as a starting point for functional studies. Analysis of pleiotropic effects could identify new susceptibility loci responsible for known comorbidities in human diseases. Examples include studies of major depression and loneliness [204], AD and diastolic blood pressure [205] and obesity and walking pace [5]. In GWAS on the volume of the human thalamus and its nuclei, LDSC was used for the discovery of significant positive correlations between the genetics of the volume of posterior nuclei and bipolar disorder, the volume of intralaminar nuclei and multiple sclerosis and the volume of the whole thalamus and Parkinson’s disease [139]. In a 2021 screen by Xicoy et al., for co-heritability between Parkinson’s disease and levels of 370 lipid species and lipid-related molecules in blood, eight specific lipid levels were found to share a significant portion of genetic architecture with Parkinson’s disease, which could have direct implications in explaining the aetiology of the disease and in practical diagnostics [206].

With the abundance of human traits available for analysis to date, it has even become possible to perform ‘reverse GWAS’ analysis, in which a plethora of traits are tested for association with a specific SNP. This analysis is called phenome-wide association, or PheWAS (Figure 1e) [207]. PheWAS of the associations of diverse complex traits in data from the UK Biobank showed that pleiotropic effects are extremely common [208]. In a practical sense, PheWAS could be used to test the pleiotropy of SNPs for a candidate medical target [156,209]. In addition, PheWAS, paired with MAGMA, could be generalised on the level of genes and gene sets [203]. This, in particular, allows researchers to test PheWAS trait enrichment in a xenobiological context with orthologous genes. For example, the GWAS signals for the phenotypes of the quality of ovine wool are, unsurprisingly, found to be associated with the human traits of hair colour and baldness [210].

In many situations, researchers are interested not only in similarity of traits, but also in causal relationships between them. There is an approach, instrumental variable (IV) analysis, that exploits the genetics of complex traits to address this type of question. In social sciences and epidemiology, when studying the causal interaction of exposure and outcome, it is often difficult to isolate them from the influence of other external factors that have not been taken into account in the model. To solve this problem, IV analysis utilises so-called instrumental variables that should be (1) associated with the exposure, (2) associated with the outcome only by means of the exposure and (3) not be associated with any confounders, including unknown ones (Figure 1f). If this is the case, it is possible to study the relationship between outcome and exposure, conditioned on the IV. The beauty of this approach is that it allows one to imply causality regardless of the existence of any unknown confounders or reverse causality. An example is an experiment that studied the causal relationship between maternal smoking and birthweight using various state cigarette taxes in the USA as an IV, in which it could be seen that cigarette taxes clearly meet all three conditions and could be used as an IV for this problem [211]. In practice, it is difficult to find IVs that meet all three conditions. In this regard, genetic factors are amazing IVs because they are perfectly random due to panmixia and depend on hardly any external factors. This application of IV analysis is called *Mendelian randomisation* [212]. For example, the rs671 SNP in the alcohol dehydrogenase gene *ALDH2* strongly affects the ability of organisms to metabolise alcohol. This SNP is common in East Asian populations, and for men, it greatly affects their average alcohol consumption. Thus, the genotypes of rs671 could be used as an IV to study the effect of alcohol consumption on, for example, blood pressure and cardiovascular diseases risk, which turned out to be quite high [213,214]. Before GWAS, the application of this experimental design was highly situational because common individual polymorphisms with strong effects like those observed for rs671 are very rare. Since GWAS has become bigger and PRS tended to explain more of the variability in a trait, PRS became more suitable for use as IV, for example, in studies of the relationships between gout, BMI, urate and triglyceride levels [208]; brain structure and depression [215]; telomere length and type II diabetes [216]; AD and various other traits [217]; and blood pressure and various cardiovascular diseases [218]. While there are important statistical problems related to exposure measurement errors [219] and assumption of absence of measurement error in SNP-exposure association [220], the major concern with genetic IV is pleiotropy, which could violate both conditions 2 and 3. However, there are a number of statistical methods aimed to alleviate this problem with GWAS data, like MR-Egger regression [221], CAUSE [222], MR-APSS [223], cML-MA [224] and others.

## 5. Bench Use of GWAS

### 5.1. Narrow-Focus Follow-Up Studies

Typically, GWAS results contain a small number of easily interpretable results, with a majority of loci lacking ‘natural’ functional interpretations. Linkage regions of GWAS can be very expansive, contain many genes or contain genes with unknown functions. Often, a GWAS linkage region does not contain genes at all if, for example, the causal variability of a trait is associated with an enhancer for a gene located far from this region. Such situations motivate further research aimed at interpreting specific GWAS signals. For example, the biological implications of a well-known genetic signal in a very large (approximately 6 million base pairs) linkage group in the MHC cluster for schizophrenia, one of the strongest signals associated with GWAS, remained a mystery for a long time. The mystery was solved, at least partially, in the 2016 work by Sekar et al., in which this association signal was linked with the variability in the gene of complement component 4 (C4) responsible for the degree of synaptic pruning during maturation of the brain [225]. In addition, the rs1421085 polymorphism in one of the FTO gene introns resides within the linkage regions for several GWAS (BMI, obesity, type II diabetes, chronotype), and CRISPR/Cas9 editing of the SNP confirmed its role in the regulation of the expression of the neighbouring IRX3 gene and in abnormal development of adipose tissue [226,227].

Defining the biological interpretation of a specific GWAS signal often involves fine-mapping of a supposed causal SNP. Experimental data could be utilised for this purpose. In a 2021 study by Guan et al., eQTL, mQTL and ATAC-seq data were used to fine-map the region of rs164748, which was identified in GWAS for estimated glomerular filtration rate (eGFR). In this study, genome editing in mice was used to demonstrate that there are at least two genes, *DPEP1* and *CHMP1A*, involved in the regulation of ferroptosis and that they surprisingly had the opposite effects on a trait [228]. The study illustrated that a GWAS LD-region could contain multiple causal genetic factors. Fine-mapping could be performed with genetic instruments that have been discussed above, such as trans-ethnic GWAS or pleiotropy analysis, which could be further enriched with experimental data. For example, analysis of genetic associations for rheumatoid arthritis in a trans-ethnic context with chromatin accessibility data allowed researchers to narrow down credible causal SNP sets [229]. An example of a study based on joint analysis of GWAS data is the beautifully designed study on bone fragility, which is known to be a symptom of type II diabetes. Analysis of the GWAS results for both traits, together with epigenetic data, made it possible to determine the common candidate rs56371916 polymorphism in the intron of the *ADCY5* gene. *ADCY5* expression in CRISPR/Cas9-edited adipocytes and osteoblasts is highly dependent on the rs56371916 alleles, which appear to be responsible for bone fragility in type II diabetes [230].

### 5.2. Interpretation of Functional Annotations Using GWAS Results

Experimental data are often processed in bioinformatics analysis using various ontology enrichment techniques. This could of course be done with GWAS data if a researcher wants to check whether, for example, ChIP-seq active chromatin peaks are colocalised with significant SNPs for a specific trait. The modern way to do this, as discussed earlier, is to use methods specialised for functional interpretation of GWAS data, like MAGMA or S-LDSC. While all of these methods were created for the interpretation of GWAS results, they could in some circumstances be used in reverse scenarios aimed to answer questions such as which complex trait genetics corresponds to a given functional annotation or which functional annotation better describes a given complex genetic trait.

This type of analysis is natural to apply to single-cell experiments, since it assists in the interpretation of revealed cell classes. For example, in a 2021 study by Sheng et al., on single-cell characterisation over different cell types in kidneys, MAGMA was applied to gene expression data and S-LDSC was applied to the scATAC-seq data [231]. Both of these analyses uncovered enrichment by GWAS results for eGFR in proximal tubules of nephrons, which mirrors the complementary result obtained with enrichment GWAS for eGFR using LDSC-SEG on expression data in proximal tubules of nephrons [232]. Using the S-LDSC approach on brain scATAC-seq data revealed that microglia are the only cell type enriched in AD GWAS results, which is in line with previous research on the disease [233]. In a 2021 study by Kupari et al., MAGMA analysis was utilised to juxtapose neuronal cell types to specific subjective locations of chronic pain [234]. In the work of Baselmans et al., stratified LDSC was used to locate brain regions associated with the genetics of generalised well-being using brain region-specific expression and DNA methylation data [54]. Such expected enrichment does not always occur; for example, in GWAS on BMI, it was shown that most of the identified genetic factors affect the level of gene expression in neuronal tissues, which is an argument for obesity being at least partially a neurological/psychiatric trait [235].

An interesting example of GWAS-informed interpretation of expression data is described in a 2017 paper by Calderon et al., which describes a method (named ‘RolyPoly’) for cell-specific enrichment in GWAS results using scRNA-seq data on a gene level. Specifically, RolyPoly gene scores for AD GWAS in the most relevant cell type (again, microglia) are correlated with test statistics of genes found in an independent differential expression experiment on AD. The result is quite impressive, as it theoretically allows us to obtain the same information from gene expression data from healthy people with AD GWAS results and from much more elaborate experiments involving collection of specific laser-microdissected brains of deceased AD patients to study differential expression [236].

In experimental biology, the causality between a gene and a phenotype could be established in direct experiments with altered and rescued gene expression via, for example, genomic editing. However, the genetic factors of complex traits are too numerous, and their effects are too small for any existing genome editing technology to be practical to use for phenotypes manifested at the level of the whole organism. There have been attempts to utilise the Mendelian randomisation approach to find causal genes linked to a trait of interest using GWAS summary statistics and eQTL data in relevant cell types. In 2019, Porcu et al., described an approach, called ‘Transcriptome-wide summary statistics-based Mendelian Randomisation approach’, TWMR, that allows the identification of potential causal links between gene expression and a trait. The utility of the method is backed by its ability to predict significant GWAS hits retrospectively and to correctly point to known causal links, for example, between *SORT1* gene expression and low-density lipoprotein using eQTL data in liver tissues [237]. It should be noted that Mendelian randomisation with GWAS/eQTL data is not straightforward because nothing prevents SNP from bypassing gene expression and influencing a trait of interest through some unknown confounder effects. For causality estimation, it is possible to employ multiple LD-independent loci within the vicinity of a gene, which could be used to distinguish mediation and pleiotropic effects, since in a true causality situation, eQTL and GWAS effects should be correlated in multiple loci. This approach is realised in the MRLocus method [238].

### 5.3. Use of PRS in Experimental Biology

Biological perspective gives us an intuition that information that is more relevant to the biology of a trait should be a better predictor; hence, use of functional data could improve or even outperform PRS-based predictions. For example, TWAS results provide a direct link between gene expression and a trait, which is tempting to use for diagnostic purposes. In a 2021 study by Pain et al., gene expression risk scores (GeRS), which are based on imputed gene expression from TWAS data, were constructed for a panel of human diseases: rheumatoid arthritis, inflammatory bowel disease, coronary artery disease, type II diabetes, height, BMI, intelligence and depression. In all cases, while GeRS explained a significant portion of heritability, they all performed worse as a trait predictor than the corresponding PRS [239]. This could be explained as follows: the gain in predictive power from aggregation of weak genetic signals into enhanced signals of functional data is outweighed by the loss of predictive power due to pleiotropy, additional non-genetic factors and pure stochasticity.

Pleiotropy seems to drastically affect the performance of methods based on individual SNPs and individual functional effects. On the other hand, polygenic scores, which integrate all genetic effects into a single number, could surprisingly be effective in informing which genes are associated with a specific trait. In 2021, Võsa et al., studied the role of distant expression–genotype interactions (trans-eQTL) in the genetics of complex traits. Notably, trans-eQTL effects are neglected in conventional eQTL-based analyses like TWAS because potential trans-eQTLs are much more abundant than traditional cis-eQTLs and attempts to identify them all individually would inevitably fail due to the dimensionality curse. The authors found that if testing for eQTL effects was limited by SNPs known to be related to a different trait, trans-eQTL effects became apparent and widespread. They further used PRS to construct expression quantitative trait scores (eQTS), in which PRS was used as a mediator for gene expression in a blood dataset for which gene expression and genotype data were simultaneously available. This allows detection of situations in which multiple weak and trans-eQTL effects converge on a single gene. The eQTS method seems to be more appropriate for the highly polygenic nature of complex traits; for example, it proved to be able to correctly detect genes for lipid metabolism using PRS for high-density lipoprotein concentration [240].

The other way to use PRS in experimental biology is to search for rare mutations relevant to a trait. The reasons why GWAS on a common disease generally does not provide suitable candidate targets for medical research include low effects, unclear genomic location due to LD and, potentially, a too-broad mechanism of action due to pleiotropy. In contrast, rare mutations are more straightforward to interpret, more prone to have large and focused effects and can be easily manipulated with conventional genetic methods. The problem is they are, by definition, rare and in general much more difficult to find and even more difficult to identify as a modificator to a trait. Usually, these mutations are found in a family genetic analysis, massive screening of trios for de novo mutations or even more massive exome-wide screenings. At the same time, candidate regions from GWAS proved to be useful as starting points for the search for meaningful rare mutations in targeted sequencing experiments focused on, for example, inflammatory bowel disease [241,242], age-related macular degeneration [243], type II diabetes [244] and rheumatoid arthritis [245,246]. The use of PRS presents a logical development in the search for rare mutations. For example, patients with schizophrenia who have clinically significant CNVs have been shown to have lower PRS [247,248]; in addition, patients with schizophrenia have lower PRS if they carry loss of function and deleterious de novo mutations [249]. In the work of Zhou et al., this hypothesis was simulated and tested on UK Biobank data to reveal that lower PRS quantiles are expected to be enriched in rare variants with large effects [250]. The use of PRS allows one to check for enrichments in rare mutations against not phenotype (which could be rare or difficult to obtain), but the PRS of the phenotype, since a large proportion of causal rare mutations is expected for individuals with low PRS. Of course, the same logic could be applied not only to rare mutations, but also to other factors like somatic mutations and epistatic, epigenetic and environmental factors.

In general, it seems possible to use PRS in experimental biology for sample stratification. Sometimes it is difficult to generate a good cell model for a trait. For example, in psychiatric genetics, it seems that the most relevant cell model for research is a foetal brain of a future psychiatric patient, which is, of course, close to impossible to obtain. Instead, stem cells from any donor could be procured with the goal of creating a relevant cell model, leveraging PRS to study a trait of interest. For example, this logic motivated a 2020 report by Dobrindt et al., on iPSC for several extreme PRS for schizophrenia [251]. In principle, the PRS stratification can be used to generate cell models of any high-level complex trait such as family income and walking pace, which could be impossible to study at the cellular level otherwise.

## 6. Conclusions

Studies of the genetics of complex traits have now reached maturity, and powerful new instruments have been developed to measure and interpret their results. These results are usually available for any interested researcher in the form of GWAS summary statistics, which allows researchers to relate complex trait genetics to a broad range of biological experiments and unlock new experimental designs in complex traits research. For instance, LDSC and other methods discussed could be used for functional data interpretation, Mendelian randomisation-based methods could help to infer causality and polygenic scores allow the direct consideration of polygenic inheritance. However, we urge avoiding overestimation of non-additive genetic effects or underestimation of pleiotropy. We hope that after reading this review, it will be clearer for experimental biologists how the results of modern genetic research can help them in their work.

## Data Availability

Not applicable.

## Abbreviations

GWAS	genome-wide association study or GWA study
SNP	single-nucleotide polymorphism
PRS	polygenic risk score
LD	linkage disequilibrium
LDSC	LD score regression
S-LDSC	stratified LD score regression
IV	instrumental variable
ROC	receiver operating characteristic
AUC	area under the receiver operating characteristic curve
eQTL	expression quantitative trait loci
TWAS	transcriptome-wide association study
eGFR	estimated glomerular filtration rate
AD	Alzheimer’s disease
